# The Influence of Serum Uric Acid on the Brain and Cognitive Dysfunction

**DOI:** 10.3389/fpsyt.2022.828476

**Published:** 2022-04-22

**Authors:** Natasa R. Mijailovic, Katarina Vesic, Milica M. Borovcanin

**Affiliations:** ^1^Department of Pharmacy, Faculty of Medical Sciences, University of Kragujevac, Kragujevac, Serbia; ^2^Department of Neurology, Faculty of Medical Sciences, University of Kragujevac, Kragujevac, Serbia; ^3^Department of Psychiatry, Faculty of Medical Sciences, University of Kragujevac, Kragujevac, Serbia

**Keywords:** uric acid, cognition, neuroprotection, neurotoxicity, oxidative stress, inflammation

## Abstract

Uric acid is commonly known for its bad reputation. However, it has been shown that uric acid may be actively involved in neurotoxicity and/or neuroprotection. These effects could be caused by oxidative stress or inflammatory processes localized in the central nervous system, but also by other somatic diseases or systemic conditions. Our interest was to summarize and link the current data on the possible role of uric acid in cognitive functioning. We also focused on the two putative molecular mechanisms related to the pathological effects of uric acid—oxidative stress and inflammatory processes. The hippocampus is a prominent anatomic localization included in expressing uric acid's potential impact on cognitive functioning. In neurodegenerative and mental disorders, uric acid could be involved in a variety of ways in etiopathogenesis and clinical presentation. Hyperuricemia is non-specifically observed more frequently in the general population and after various somatic illnesses. There is increasing evidence to support the hypothesis that hyperuricemia may be beneficial for cognitive functioning because of its antioxidant effects but may also be a potential risk factor for cognitive dysfunction, in part because of increased inflammatory activity. In this context, gender specificities must also be considered.

## Introduction

Uric acid (UA) is the final oxidation product of adenine and guanine metabolism (1). It is formed from these exogenous purines and endogenously from damaged, dying and dead cells (2). In humans, an enzyme called urate oxidase loses its functional activity so that further oxidation of UA is no longer possible. Consequently, humans must cope with much higher levels of UA compared to other mammals (3). UA is generally known for its bad reputation. The fact that about 90% of UA filtered in renal glomeruli is reabsorbed and that humans maintain high levels of UA raised the idea that UA should be considered not only as a metabolic waste but also as a molecule with important physiological activity. The beneficial effects of UA were proposed by Kellog and Fridovich (4) and further explored and developed by Ames et al. (5) three decades ago. *In vitro* experiments showed that UA is a potent scavenger of singlet oxygen, peroxyl radicals (RO${}_{2}^{{}^{\circ}}$), and hydroxyl radicals (5). It also protects the cell from oxidative damage by chelating metal ions (6) and acting as a specific inhibitor of radicals generated by the decomposition of peroxynitrite (ONOO^−^) (7). Because of these effects, UA is considered a very potent free radical scavenger, accounting for more than half of the antioxidant capacity of plasma (8). On the other hand, many epidemiological and experimental data show the oxidative potential of UA. Various cells, after being exposed to UA, generate reactive oxygen species (9). UA, as a pro-oxidant, can decrease nitric oxide (NO) production, induce lipid peroxidation, and interact with peroxynitrite to generate free radicals (9). In recent years, the mechanisms by which UA mediates inflammation have attracted the interest of scientists. UA has been found to contribute importantly to immune responses even in the absence of microbial stimulation (10).

UA (2,6,8 trioxypurine—C_5_H_4_N_4_O_3_) is a heterocyclic organic compound that is the end product of the oxidation of two purine nucleic acids, adenine and guanine. The enzymatic pathway for the degradation of purines is complex and involves numerous enzymes. Briefly, adenosine monophosphate (AMP) is converted to inosine, while guanine monophosphate (GMP) is converted to guanosine by nucleotidase. The nucleosides, inosine and guanosine, are further converted by purine nucleoside phosphorylase (PNP) to the purine bases hypoxanthine and guanine, respectively. Hypoxanthine is then oxidized to xanthine by xanthine-oxidase (XO) and guanine is deaminated to form xanthine by guanine deaminase. Xanthine is again oxidized by xanthine oxidase to form the final product, UA (11). Being a weak acid with a high dissociation constant, UA circulates in plasma (pH 7.4) predominantly in the form of urate (98%), a monovalent sodium salt (1). UA is mainly formed in the liver, intestine and vascular endothelium from endogenous (nucleoproteins) and exogenous (dietary proteins) precursor proteins (2). Approximately two-thirds of the UA load (65–75%) is excreted by the kidneys, while the gastrointestinal tract eliminates one-third (25–35%) (12). Most of the serum UA is freely filtered in kidney glomeruli, and about 90% of the filtered UA is reabsorbed, while only 10% is excreted in the urine (1).

The UA serum level is the result of a balance between dietary purine intake, xanthine oxidase activity, and renal UA excretion (13). When the balance is disturbed, hyperuricemia or hypouricemia occurs. Hyperuricemia has been arbitrarily defined as a value >7 mg/dl in men and >6.5 mg/dl in women, while hypouricemia is defined as a serum urate concentration ≤ 2 mg/dl (14). Numerous epidemiological studies showed elevated UA levels in patients with gout (15), chronic kidney disease (16), cardiovascular diseases (17), metabolic syndrome and obesity (18), confirming its role as a risk factor and useful marker for prediction of progression and outcome in these diseases.

Hyperuricemia has been studied as a possible driving force in the development of intelligence in primates (19). The presence of hyperuricemia has been shown to confer an evolutionary advantage through greater stimulation of the cerebral cortex (20), which could be attributed to its structural similarity to the known psychostimulant caffeine (19), but has also led to longer life in hominids due to its antioxidant effects (21). These UA metabolic properties may have allowed humans to develop higher brain mass (in terms of volume), better intellectual performance (22), and possibly evolutionary supremacy (20) compared with other mammals. The relationship between hyperuricemia and intellectual activity was then established in different population samples (23, 24). The importance of this relationship has been confirmed at the level of cortical stimulation and/or facilitation of learning processes (22).

Epidemiological studies showed that hyperuricemia is increasing worldwide (25). All these features of elevated UA levels were no longer beneficial but rather became risk factors in modern humans, suggesting that UA plays an important pathogenic role in “diseases of civilization” (26). A recent confluence of biochemical, epidemiological and clinical data has pointed to the far-reaching neuroprotective potential of this endogenous antioxidant but also highlighted its role in inflammatory processes. Although a relatively simple substance, the implications of UA's complex effects on health and disease must be considered.

Understanding the mechanisms by which high UA levels affect neuroplasticity and cognitive functioning could provide a potential therapeutic approach to counteract diseases associated with hyperuricemia. Considering the complexity of the human organism, none of the metabolites, including UA, can be considered one-sidedly. In this article, we attempt to elucidate the role of UA in cognitive functioning based on its involvement in oxidative stress and inflammatory processes.

## Methodology

This narrative review was performed by an exhaustive electronic search of the PubMed and Web of Science databases using the terms “uric acid” and “cognition”; “uric acid” and “oxidative stress” “uric acid” and “neuroinflammation;” “uric acid” and “neuroprotection;” “uric acid” and “neurotoxicity”. There was no restriction on the year of publication. We searched for studies published in English, but there were no regional restrictions. We did not pre-specify a preferred study methodology, so there was no restriction on a particular study design. Experimental studies, randomized or non-randomized clinical trials, cohort studies, and case-control studies were considered. We did not limit the assessment of cognitive functioning to a particular test or specify the method of serum UA (sUA) measurement. Abstracts of potentially relevant titles were assessed, and the full text of potentially eligible studies was reviewed. We performed a forward and backward search for relevant papers and repeated this process until no new titles were found. Letters, comments, editorials, practice guidelines, conference proceedings, theses, case studies and unpublished data were excluded.

## Uric Acid and Oxidative Stress

**Figure 1:**
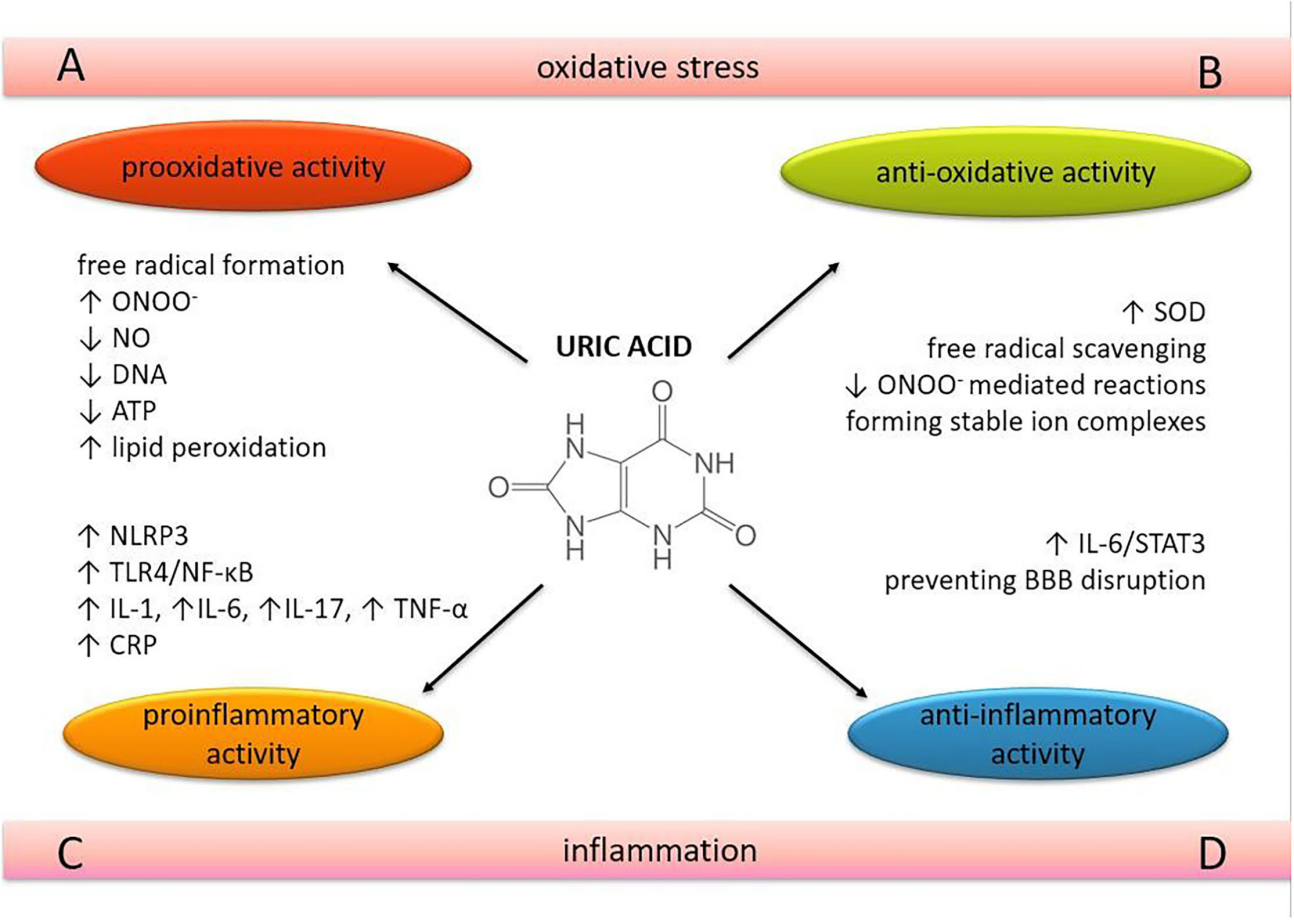
The role of uric acid in oxidative stress and neuroinflammation. The dual nature of uric acid in terms of oxidative and inflammatory processes in brain tissue. ONOO^−^, peroxynitrite; NO, nitric oxide; SOD, superoxide dismutase; DNA, deoxyibonucleic acid; oxidative **(A,B)** and inflammatory processes. **(C,D)** in brain tissue. ATP, adenosine 5'-triphosphate; NLRP3, nucleotide-binding and oligomerization domain-like receptor protein 3; TLR4, Toll-like receptor 4; NF-κB, nuclear factor kappa-light-chain-enhancer of activated B cells; IL, interleukin; TNF-α, tumor necrosis factor-alpha; CRP, C-reactive protein; STAT 3, signal transducer and activator of transcription 3; BBB, blood-brain barrier.

UA acts as a pro-oxidant by increasing free radical production, causing inflammation, and altering the production of NO (27). UA can become a pro-oxidant by forming radicals in reactions with other various oxidants (28), including its relevant interaction with peroxynitrite (27, 29). These radicals predominantly target lipids, low-density lipoprotein (LDL), and membranes rather than other cellular components. At the same time, the hydrophobic environment created by lipids is unfavorable for UA to exert its antioxidative properties. UA cannot scavenge lipophilic radicals and cannot break the radical chain propagation within lipid membranes (30). UA can oxidize LDL in the presence of copper ions (Cu^+^ and Cu^++^) and lipid hydroperoxidases (31). UA decreases the bioavailability of NO and inhibits cell migration and proliferation in endothelial cells, mediated in part by C-reactive protein (CRP) expression and oxidative stress (32). It also decreases mitochondrial deoxyribonucleic acid (DNA) contents and intracellular adenosine triphosphate (ATP) concentrations associated with reactive oxygen species (ROS) production (33) (Figure 1A). However, the reaction of UA with peroxynitrite can also generate radicals, consistent with the ability of UA to become pro-oxidant under various circumstances (34).

Oxidative stress has been shown to occur in various cells exposed to UA, such as vascular endothelial cells (32, 33, 35), adipocytes (36), renal tubular cells (37), hepatocytes (38), etc. The high oxygen consumption in neurons leads to the formation of an excessive amount of ROS in the central nervous system (CNS). Compared with other organs, the brain has a lower antioxidant capacity, which makes it particularly vulnerable to oxidative stress (39). The lipid structure of neuronal membranes with unsaturated fatty acids makes neurons extremely sensitive to lipid peroxidation (40). Oxidative damage in the CNS is the result of oxidation and nitration of proteins, lipids, and DNA, leading to necrosis and apoptosis of neuronal cells (41).

UA is a very potent free radical scavenger and is considered one of the most important antioxidants in human plasma (5) (Figure 1B). It has been suggested that UA may exert neuroprotective effects because of its antioxidant properties. The neuroprotective effect of this purine metabolite was demonstrated in cultured rat hippocampal neurons exposed to excitatory and metabolic toxicity. It also resulted in stabilization of calcium homeostasis and preservation of mitochondrial function (42). Serum UA levels have been shown to have a significant positive correlation with total serum antioxidant capacity in healthy human volunteers after acute administration (41, 43) and also in hypoxia-induced conditions (44). Superoxide dismutase (SOD) is an antioxidant enzyme that scavenges superoxide anion (O${}_{2}^{-}$) by converting this free radical into oxygen (O_2_) and hydrogen peroxide (H_2_O_2_). Hink et al. demonstrated that UA effectively preserves and enhances extracellular SOD activity in mice at concentrations approaching physiological levels in humans (45). Removal of O${}_{2}^{-}$ prevents its reaction with NO, thus blocking the formation of peroxynitrite (ONOO^−^), a very potent oxidant implicated in the pathogenesis of several CNS diseases. Peroxynitrite can interact with almost all cellular structures, causing severe cellular damage (46, 47). Squadrito et al. have shown that UA cannot scavenge ONOO^−^ directly but acts as a specific inhibitor of radicals such as CO${}_{3}^{-}$ and NO_2_, which are formed when ONOO^−^ reacts with CO_2_ (48). The protective effect of UA against ONOO^−^ was confirmed in the experimental autoimmune encephalomyelitis (EAE) model in mice, which is a model of multiple sclerosis (MS) (49). In mice with developed EAE, exogenously administered UA penetrated the already compromised blood-brain barrier (BBB) and blocked peroxynitrite (ONOO^−^) mediated tyrosine nitration and apoptotic cell death in inflamed areas of the spinal cord tissue.

UA, as a selective inhibitor of certain peroxynitrite-mediated reactions, blocked the toxic effects of peroxynitrite on primary spinal cord neurons *in vitro* in a dose-dependent manner and also inhibited both the decline in mitochondrial respiration and the enhanced release of lactate dehydrogenase (LDH) (50). In a mouse model of spinal cord injury (SCI), treatment with UA prevented nitrotyrosine formation, lipid peroxidation, and neutrophil infiltration into spinal cord tissue and significantly improved locomotor dysfunction in mice (50). These results support the possibility that elevating UA levels may provide a therapeutic approach for the treatment of SCI as well as other neurological diseases with a peroxynitrite-mediated pathological substrate.

## Uric Acid and Inflammation

UA is thought to have a pro-inflammatory effect by triggering interleukin (IL)-1β-mediated inflammation via activation of the nucleotide-binding and oligomerization domain (NOD)-like receptor protein (NLRP) 3 inflammasome, a multimolecular complex whose activation appears to play a central role in many pathological inflammatory conditions (51, 52). It also induces the expression of CRP in human vascular cells (32). Epidemiological studies have shown that UA is positively associated with several pro-inflammatory markers such as CRP, white blood cell count, IL-6 and tumor necrosis factor-alpha (TNF-α), and predicts an increase in their levels over a 3-year follow-up (53, 54). A recent randomized, double-blind, placebo-controlled pilot study revealed the positive correlation between serum UA levels and IL-6, IL-17, and TNF-α, suggesting that xanthine oxidase inhibitors reduce serum UA levels but also the levels of these cytokines in patients with gout (55) (Figure 1A). A positive relationship between serum UA and acute-phase reactants such as CRP, fibrinogen, ferritin, and complement C3 was confirmed in a dose-dependent manner, also suggesting that UA induces the pro-inflammatory effect through the nuclear factor kappa-light-chain-enhancer of activated B cells (NF-κB) signaling pathway (56) (Figure 1C).

Elevated UA levels induced by a high-UA diet (HUAD) triggered the expression of pro-inflammatory cytokines, activated the Toll-like receptor 4 (TLR4)/NF-κB pathway, and increased gliosis in the hippocampus (57) and mediobasal hypothalamus of Wistar rats (58). Furthermore, serum UA was able to cross the BBB and act as a potent inflammatory stimulus (57, 58). Some authors found a linear correlation between serum UA levels and UA levels from cerebrospinal fluid (CSF) and confirmed that BBB impairment was associated with higher CSF levels of UA (59). TLR signaling pathways culminate in the activation of the transcription factor NF-κB, which controls the expression of an array of inflammatory cytokine genes (60). In addition, the activation of the TLR4/NF-κB signaling pathway also occurs in other pathological states which are induced by UA, such as pancreatic β-cell death (61) and renal tubules (62). These results indicate that the pathogenic effect of UA may be manifested by inflammation in the hippocampus, suggesting NF-κB activation as an important signaling pathway. Aliena-Valero et al. demonstrated that exogenous administration of UA increases IL-6 levels and plays a neuroprotective role through the activation of the IL-6/signal transducer and activator of transcription 3 (STAT3) signaling pathway, which in turn leads to modulation of relevant mediators of oxidative stress, neuroinflammation, and apoptotic cell death in the brain (63).

The protective role of UA has also been observed in CNS inflammatory processes. In the EAE model, exogenous treatment with UA prevented disruption of BBB integrity and reduced its permeability to inflammatory cells (49) (Figure 1D). Pre-treatment with UA attenuated meningeal inflammation, BBB, and intracranial hypertension in a dose-dependent manner in the adult rat pneumococcal meningitis model. As UA levels increased to approach levels found in humans, the severity of inflammation decreased as a function of UA concentration (64).

## Uric Acid and Cognitive Functioning

Cognition as a higher brain function consists of major cognitive domains: memory, attention, language, executive functions and visuospatial functions (65). All of these domains can be affected and impaired by certain diseases, processes, or toxins, resulting in cognitive dysfunction (65). Cognitive impairment is a chronic neurodegenerative condition characterized by poor learning and memory (66). Cognitive impairment can be a consequence of the physiological aging process (67, 68), but it can also accompany neurodegenerative (69) and neuropsychiatric disorders (70, 71). It is well-established that oxidative stress and inflammation are important pathogenic mechanisms that lie in the background of these conditions (72–77).

As a potent antioxidant in the human body, but also as a mediator in inflammatory processes, UA is the subject of increasing research focused on its influence on cognitive functioning (summarized in Table 1). Although the impact of UA on cognitive functions is undoubtedly confirmed, the exact mechanisms by which cognitive changes occur are not fully understood.

**Table 1:** Correlation between serum uric acid levels (sUA) and cognitive functioning (CogF) in various study populations.

**↑sUA **~**↓CogF**	**↑sUA **~**↑CogF**	**↓sUA **~**↓CogF**
- Subjects aged 20–80 years (78) - Pharmacology untreated young elderly adults (79) - Elderly adults (80) - Subjects mean age 62 years (81) - Community-dwelling older persons (82) - Older man (83) - Healthy older women (84) - Older adults with cardiovascular disease (85) - Patients with chronic kidney disease (86–88) - Patients with chronic heart failure (89) - Cognitively healthy man adults (90) - Healthy older people mean age 72 (91) - Elderly hemodialysis subjects (92) - Post-stroke patients (93) - Male patients with ischaemic stroke and transient ischaemic attack (94)	- Middle-Aged and older people (95) - Subjects aged 50–74 years (96) - Subjects aged 55 years and over (97) - Very old people (age 90–108 years old) (98) - Females with MCI, apoE4 carriers (99) - Healthy middle-aged man population (100) - Oldest-Old aged 80 years and older (101) - Older adults aged 65 and over (102) - Patients with PD (103, 104) - Patients with ALS (105–107) - Patients with HD (108) - Patients with AD (109)	- Men aged 45–74 with high vascular burden (110) - Post-stroke patients (93) - Patients with PD (111) - Patients with depression (112)

*↑, elevation or improvement; ↓, decreasing or declining; MCI, mild cognitive impairment; PD, Parkinson's disease; ALS, amyotrophic lateral sclerosis; HD, Huntington's disease; AD, Alzheimer's disease*.

**Figure 2:**
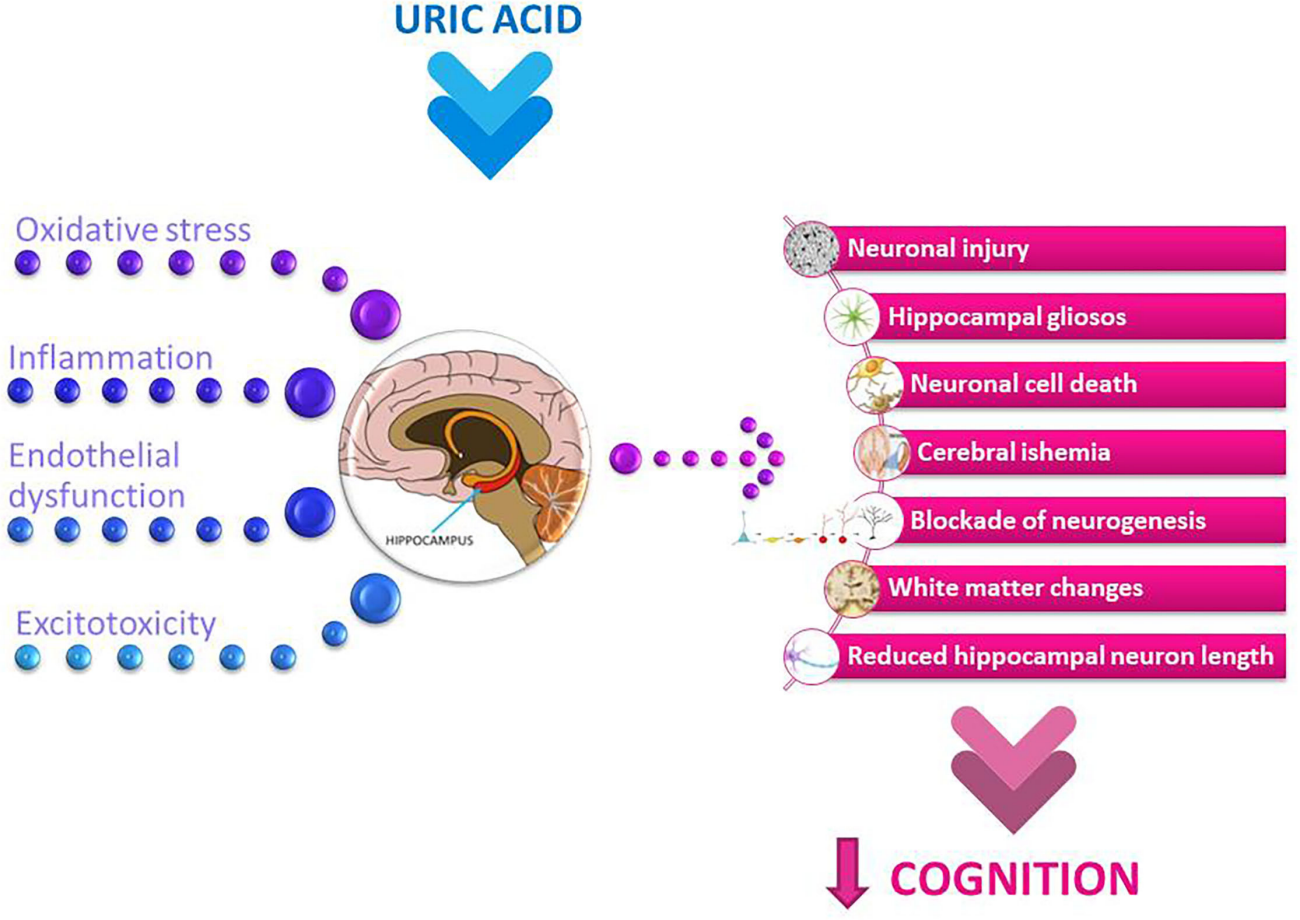
Potential mechanisms involved in uric acid-related cognitive dysfunction. The summary of the main pathological mechanisms of uric acid, such as oxidative stress and neuroinflammation, along with endothelial dysfunction and excitotoxicity, which may collectively affect neuronal and brain function and further implicate uric acid-related cognitive decline.

The neuroanatomy of cognition should be considered in more detail in this context. The hippocampus is the brain region that plays a critical role in learning and memory (presented in the center of Figure 2). Hippocampal dysfunction can alter cognitive abilities (113). Inflammation of the hippocampus has been associated with various neurological dysfunctions (114, 115). The inflammatory responses also lead to neuronal death and blockade of neurogenesis, which in turn leads to cognitive impairment (116, 117).

In physiological settings, UA levels have been measured in the serum and its values were established to be in the range of 3.5 and 7.2 mg/dL in adult males and postmenopausal women and between 2.6 and 6.0 mg/dL in premenopausal women (118). Under physiological conditions, the brain relies relatively little on UA for antioxidant defense because UA molecules cannot leak through an intact BBB (119). The relation between UA serum levels and CSF levels represents a crucial aspect in assessing the influence of UA on brain tissue. In healthy subjects, CSF UA levels are about ten times lower than serum levels (120). Examining CSF metabolite in a healthy population sample, Reavis et al. (121) recently found that men have higher CSF UA levels than women [median (25th−75th) 5,353.71 ng/mL (4,041.17–7,102.65) vs. 9,008.48 ng/mL (7,033.92–11,906.72), *p* < 0.0001]. BBB destruction is thought to play an important role in neuroinflammation and oxidative stress (122). Previous studies have shown that the concentrations of CSF UA in patients with an impaired BBB depend partly on serum UA concentrations and partly on the balance between production and consumption in the CNS (123). Some authors have proposed the CSF-to-plasma UA ratio as a marker of BBB integrity (124). The urate transporter URAT1, expressed on cilia and the apical surface of ventricular ependymal cells lining the wall of the ventricles that separates CSF and brain tissue, may also represent a novel UA transport mechanism involved in the regulation of UA homeostasis in the brain (125). Desideri et al. provided the evidence that UA could exert detrimental effects on brain structure and function by directly influencing the viability of neuronal cells and their ability to establish synaptic connections in the *in vitro* model of Alzheimer's dementia (AD), depending on the levels of exposure of cells to UA (126). This effect of UA was observed starting from the dose of 40 mM, while lower UA concentration did not significantly influence cell biology, suggesting a dose-dependent effect of this purine metabolite. The reduction of cells viability under UA exposure was observed starting from a dose that could be achieved in CSF in a condition of mild hyperuricemia (i.e., 400 mM) (118). Intraperitoneal injection of UA was found to elevate both plasma and brain urate levels by 55 and 36.8%, respectively, in rats (127). In recent years, there is increasing evidence of positive correlations between CSF UA and sUA levels in patients with neuroinflammatory and neurodegenerative diseases, supporting the hypothesis of a strong influence of UA on the brain and cognition (59, 123, 128). In the presence of hyperuricemia, the diffusion of UA through the BBB could increase the concentrations of UA in CSF to the levels that might exert detrimental effects on cells biology by promoting the onset and/or progression of neuronal damage, further leading to cognitive impairment. It has been shown that each μmol/L increase in plasma UA was associated with about a 5% increase in CSF UA in patients with mild cognitive impairment (59).

Shao et al. showed that systemic hyperuricemia, induced by HUAD in rats over a 12-week period resulted in cognitive dysfunction manifested by decreased spatial learning and memory (57). TLR4 activation can reduce hippocampal pyramidal neuron dendrite length and impair hippocampal-dependent spatial reference memory in an inflammation-dependent manner (129), thus suggesting the potential for TLR4 activation by UA which lead to cognitive impairment. Decreased SOD activity was also detected in the hippocampal tissue of HUAD rats, suggesting pro-oxidant activity of UA. The concentration-dependent correlation between serum levels of UA and hippocampal gliosis has also been confirmed in humans (57). Hypothesizing that the effects of UA on cognition may be related to its concentration and exposure period, Tian et al. explored the effects of long-term elevated serum UA level on cognitive function and hippocampus. This UA elevation induced by HUAD during 48 weeks was significantly associated with the risk of cognitive impairment (130). Elevated UA levels induced oxidative stress and increased the expression of TNF-α and amyloid beta peptide (Aβ) in the rat's hippocampus, suggesting that both oxidative stress and inflammation could mediate the pathogenesis of cognitive impairment induced by UA (130). This study also suggests that the detrimental effects of higher UA levels on cognitive functioning are likely to become apparent only above a certain serum UA concentration. In this review, we have addressed the problems of the possible different impacts of this molecule on cognitive functioning, especially in the context of oxidative and inflammatory changes, and further tried to enlighten its diverse impact of cognition in various neuropsychiatric disorders and regarding somatic functioning in animal models and human studies.

### Uric Acid and Cognitive Abilities in an Aging Population

The results of epidemiological studies on the relationship between UA and cognition are conflicting. The study examining the cognitive decline in the population of healthy older women showed that elevated UA levels were associated with poorer working memory and slower manual speed but not with global cognitive functioning, learning/memory, verbal fluency or visuo-constructional functions (84). In the study of elderly adults with mildly elevated UA levels, poorer working and verbal memory were observed compared with those with low-intermediate UA concentrations (80). It has also been shown that even mildly elevated UA levels can lead to both structural and functional brain changes. The results of the Invecchiare in Chianti (InCHIANTI) cross-sectional study suggest a positive association between high circulating levels of UA and the presence of dementia syndrome (82). A positive correlation between circulating UA levels and cognitive decline was demonstrated in a cohort of pharmacologically untreated young elderly subjects (79). In the Rotterdam Scan Study, hyperuricemic patients exhibited white matter atrophy compared to normouremic subjects. This structural change was followed by a deterioration in cognitive abilities, as evidenced by poorer information-processing speed and decreased executive functionality (81). Beydoun et al. showed that in older men, a significant increase in serum UA levels was associated with faster cognitive decline over time in a visual memory/visuo-construction ability test (83). Elevated serum UA levels were associated with changes in spontaneous brain activities and also followed by lower learning/memory and attention/executive functions (78). Lower neuropsychological assessment scores were notably detected in word fluency tests and number connection tests and were observed in males with pre-hyperuricemia and hyperuricemia (78). Because the cognitive changes occurred before hyperuricemia, this finding may be relevant to the clinical management of patients with pre-hyperuricemia and hyperuricemia. These results also suggest that the changes in cognitive functions affected by the different serum UA levels are gender-specific. This study demonstrated that the changes in spontaneous brain activity occurred mainly in the pallidum and putamen, which were correlated with scores of verbal fluency tests and number connection tests. The pallidum and putamen are the structures that make up the basal ganglia, which are involved in motor control and learning and in the selection and activation of cognitive, executive, and emotional programs (131). The gender-related effect has also been observed in some other studies. A study of 1,451 cognitively healthy adults found that elevated baseline sUA was related to decreased attention and visuospatial abilities in males. There were no noticeable findings in females (90). In a large cohort of 1,598 healthy older people, mean age 72 years, with a follow-up of 12 years, Latourte et al. found an increased risk of developing dementia in those with high sUA levels, even after multiple adjustment (91). A strong association was found with vascular or mixed dementia and no significant association with AD. The authors found no significant association between sUA levels and magnetic resonance imaging markers of cerebrovascular disease or hippocampal volume (91). Elevated UA levels could contribute to endothelial dysfunction (132) and subsequent white matter lesions (85) by reducing the availability of NO in the brain, which in turn leads to poorer cognitive performance. In addition, UA could also contribute to endothelial dysfunction through its pro-oxidant properties (133) (see in Figure 2).

In contrary to previous observations, a large prospective population-based cohort study of 4,618 participants aged 55 years and older showed that elevated UA levels were associated with a decreased risk of dementia. Participants without dementia who were followed up later in life and developed hyperuricemia also had better cognitive performance across all cognitive domains assessed in the study but after adjustment for several cardiovascular risk factors (97). Elevated serum UA levels adversely affected subjects with normal cognition, whereas a protective trend was observed in individuals with cognitive impairment. Interestingly, higher sUA levels were associated with a slower decline in cognitive scores and brain metabolism in females with mild cognitive impairment (MCI), and this effect was found in apolipoprotein E4 carriers but not in non-carriers (99). The cohort study of very old people (age 90–108 years) showed that higher sUA levels were associated with a lower risk of cognitive impairment, but only in men (98). This gender-dependent effect was also suggested in a cross-sectional analysis from the Brazilian Longitudinal Study of Adult Health (ELSA-Brazil) cohort (100). Similar results were obtained from studies among Chinese older adults examining the association between blood UA levels and risk for MCI, suggesting a protective role of high blood UA levels. The findings highlight the potential of managing UA in daily life for preserving cognitive abilities in later life (101, 102). High circulating UA levels correlated positively with improved muscle function and cognitive performance in elderly subjects (96). In a prospective study, elevated baseline UA levels were associated with subsequently enhanced cognitive performance, even in the specific cognitive domain (95).

Both vascular pathology and oxidative stress have been associated with increased risk of dementia and cognitive impairment (134, 135). The results of a recent study by Sun et al., demonstrated that serum UA levels were significantly higher in the PSCI group than in the non-PSCI group, suggesting that serum UA levels may serve as a predictive factor for PSCI (93). In the analysis of data from the Impairment of Cognition and Sleep (ICONS) study, both low and high sUA levels were associated with an elevated incidence of PSCI in males but not in females (94). Although UA has potent antioxidant properties, it can accelerate the oxidative stress reaction under certain pathological conditions, such as ischaemia (136, 137).

Several recent systematic reviews have carefully examined the data on sUA and the relationship with dementia/cognition and provided a more comprehensive synthesis of the evidence. The systematic review by Khan and colleagues showed that in 31 studies using mostly case-control data, sUA was lower in dementia patients compared with control subjects without dementia (138). This review concluded that the relationship between sUA and dementia/cognitive impairment was not consistent across dementia groups, with an apparent association for AD and Parkinson's-disease-related dementia (PDD), but not in cases of mixed dementia or Vascular Dementia (VaD). There was no correlation between scores on Mini-Mental State Examination (MMSE) and sUA level, except in patients with PDD. Similar results were provided by the meta-analysis of cohort studies by Pan et al. (139). Another systematic review assessed the association between sUA and AD (140). Based on 11 case-control studies with 2,708 participants, the sUA levels were not significantly different between patients with AD and healthy controls. Thus, on the basis of these systematic reviews, there is no convincing evidence to date that higher sUA levels are associated with a lower risk of dementia, except possibly in PDD.

### Uric Acid and Cognitive Abilities in Accompanied Somatic States

Elevated UA levels are an important risk factor for chronic kidney disease (CKD), and numerous studies have shown that these patients are at higher risk for cognitive impairment (86–88). It is also well-established that cerebrovascular lesions are an important risk factor for the development of cognitive decline in CKD patients (141, 142) and that UA plays an important role in these lesions (143, 144). Moreover, direct neuronal injury by uremic toxins can significantly alter cognitive functions in patients at all stages of CKD (87, 145, 146) (summarized in Figure 2). The negative correlation between sUA and MMSE scores was also found in elderly patients receiving maintenance haemodialysis. This correlation was independent of demographic and clinical characteristics (92).

The association between UA and subsequent cognitive performance in patients that carry a high vascular burden showed that low UA levels were associated with poorer cognitive performance, manifested by lower global cognitive scores, memory scores, executive scores, and visuospatial scores (110). A stronger UA effect on cognitive performance was found in older patients (>65 years old), with a significant age interaction for global cognitive, executive, and attention scores. The main finding of this study is that among men with long-lasting cardiovascular diseases and a high vascular burden, lower UA levels were associated with poorer cognitive functions assessed a decade later (110). Higher serum UA levels were independently associated with poorer cognitive performance in chronic heart failure patients (89). Furthermore, these UA effects were manifested in men but not in women.

### Uric Acid and Neurodegeneration

The potential contribution of UA to cognitive reserve could be attributed to its potent antioxidant properties. Euser et al. examined the association between serum UA and lower dementia risk and better cognitive function later in life in a large prospective population-based cohort study over an 11-year follow-up period (97). Higher levels of UA were associated with lower dementia risk and better cognitive function in later life. UA has revealed neuroprotective effects after experimental cerebral ischemia (42). These findings support the central role of oxyradicals in excitotoxic and ischemic neuronal injury and suggest a potential therapeutic use of UA in ischemic stroke (refer to Figure 2). Neurological impairment at stroke onset and final infarction size at follow-up were inversely related to the concentration of UA (147). Recent studies on the recovery of cognitive function after stroke by the use of UA may also indicate its possible role in the formation of a cognitive reserve (97).

There is growing evidence that UA may exert neuroprotective properties by suppressing neuroinflammation and inhibiting oxidative stress in neurodegenerative disorders (148, 149). In the experimental model of MS, exogenous treatment with UA prevented disruption of BBB integrity, reduced its permeability to inflammatory cells, decreased oxidative stress, and promoted the survival rate (49). Clinical trials consistently suggest that higher serum UA levels are related to a slower progression of Parkinson's disease (PD) (103, 104). PD patients with cognitive dysfunction also have lower serum levels of UA compared to those without cognitive dysfunction (111). The controlled longitudinal study that examined the evolution of cognitive changes and the prognostic value of the UA levels on cognition in the PD-patient cohort demonstrated that the level of both plasma and sUA remained stable over the 3-year period with subtle cognitive changes (150). Given that UA may have a neuroprotective effect in PD, maintaining or even increasing the sUA levels would be beneficial for PD-patients. This study also suggests that it is important to keep body weight and diet stable to avoid fluctuations in UA levels. Recent epidemiological studies showed decreased levels of UA in amyotrophic lateral sclerosis (ALS) patients compared to matched controls and subsequently linked higher UA baseline levels with slower progression and prolonged survival (105–107). In Huntington's disease, functional decline was negatively correlated with UA levels (108). The inverse association between serum UA and AD risk was confirmed in the meta-analysis by Du et al. (109). Patients with depression seem to have significantly lower serum UA levels compared to patients with delirium, dementia, amnesia, and other cognitive disorders (112). The decrease in serum UA levels was related to the antimanic, anticonvulsant, and antiagressive effects of lithium and allopurinol (151). UA levels were also decreased in subjects with first-episode psychosis (152), and further reduced plasma UA levels suggest a defect in the antioxidant defense system in schizophrenia (153). Numerous studies have shown that serum UA levels were lower or tended to decrease in patients with neurodegenerative and mental disorders. Increased serum UA levels could reduce the risk of onset and slow the progression of cognitive decline, thus confirming the hypothesis of a protective role of UA in these disorders (Figure 1B). Opposite to these results, recent research by Borovcanin et al. pointed to the correlation of sUA levels with negative symptoms in patients with schizophrenia after acute treatment, especially important when considering that negative symptoms and cognitive deficits in schizophrenia share many features (154).

## Conclusion

Based on the results of experimental and clinical studies, UA seems to play a dual role as a pro- and antioxidant. The balance between the two effects reflects a very complex interplay of factors that include the concentration of UA, the nature and concentration of free radicals, the presence and concentration of other antioxidant molecules, and the various cascades involved (155). However, recent studies suggest that the antioxidant properties of UA are not solely responsible for its beneficial effects in the CNS. UA has been recognized as an important metabolite in protecting spinal cord neurons from glutamate-induced toxicity (156). UA is also thought to have beneficial effects within the normal range, whereas detrimental effects are more likely to occur in hyperuricemia.

Recently, however, there has been a growing body of evidence from clinical and basic research supporting the hypothesis that hyperuricemia, in part through increased inflammatory activity, may be a potential risk factor for cognitive dysfunction. Taking these lines of evidence together, UA appears to exert a protective effect on brain tissue and neurons during the initial stage of elevated UA levels, primarily through its potent antioxidant activity, but long-term elevation appears to trigger an inflammatory response that leads to brain tissue damage. Thus, UA metabolism may be a so-called double-edged sword in terms of the inflammatory and/or oxidative responses it induces in brain tissue, although, its harmful effects appear to outweigh the benefits of UA in most cases.

Although numerous factors contributing to cognitive impairment have been identified to date, UA appears to be an important participant in the onset and/or progression of cognitive decline in various disorders. There is still conflicting evidence about UA's pathophysiological role and its clinical significance in influencing cognitive dysfunction. This may be partly explained by UA's dual nature and different properties, but also by a variety of distinct pathologies that can lead to many constellations of cognitive domain's dysfunctions.

## Future Perspectives

The UA impact on cognitive abilities may have a long evolution course, suggesting that the effects of UA on cognition should be explored in the terms of long, chronic exposure. The more informative study design would be a prospective long-term follow-up cohort study with a relatively large sample of older adults and measurement of sUA fluctuations concurrently with cognitive testing. Cognitive functions should be assessed with a wider range of domain-specific neurological tests. This would allow us to understand global patterns of cognitive fluctuation over time. In this context, it would also be important to investigate accompanying neuroanatomical and neurophysiological changes. Identification of modifiable risk factors is also important, as this will provide greater insight into pathophysiology, risk stratification and potential interventions. Collection of data on dietary habits, medication and comorbidity is necessary to fully exclude the influence of confounding factors. Further research on these complex topics is needed to help design and implement interventions to preserve cognitive capacities in health and various diseases.

## Author Contributions

MB presented the idea and initial structuration of this review article. NM and KV drew a figure and a table. All authors have searched the literature and given some new insights into specific fields of their competencies, read, discussed, and accepted responsibility for the entire content of this submitted manuscript and approved its submission.

## Funding

This work was supported by the Ministry of Science and Technological Development of the Republic of Serbia, No. 175069 and the Faculty of Medical Sciences, University of Kragujevac, No. JP 03/16.

## Conflict of Interest

The authors declare that the research was conducted in the absence of any commercial or financial relationships that could be construed as a potential conflict of interest.

## Publisher's Note

All claims expressed in this article are solely those of the authors and do not necessarily represent those of their affiliated organizations, or those of the publisher, the editors and the reviewers. Any product that may be evaluated in this article, or claim that may be made by its manufacturer, is not guaranteed or endorsed by the publisher.
